# Research on Cleft Lip and Palate: What Is New?

**DOI:** 10.3390/children11010025

**Published:** 2023-12-25

**Authors:** Eloá Cristina Passucci Ambrosio, Paula Karine Jorge, Cleide Felício Carvalho Carrara, Maria Aparecida Andrade Moreira Machado, Thais Marchini Oliveira

**Affiliations:** 1 Hospital for Rehabilitation of Craniofacial Anomalies, University of São Paulo, Rua Silvio Marchione 3-20, Bauru 17012-900, SP, Brazil; eloacpambrosio@usp.br (E.C.P.A.);; 2 Department of Pediatric Dentistry, Orthodontics and Public Health, Bauru School of Dentistry, University of São Paulo, Alameda Dr. Octávio Pinheiro Brisolla 9-75, Bauru 17012-901, SP, Brazil

## 1. Introduction

Craniofacial development begins during the fourth week of intrauterine life (IUL). It is a complex process that can be influenced by genetic and/or environmental interactions in which neural crest cells play an essential role in craniofacial morphological formation [1,2]. However, the interruption of embryogenesis between the 4th and 12th weeks of IUL can lead to errors in the fusion of facial prominences, resulting in cleft lip and palate [1,2]. These congenital craniofacial anomalies are common [3], but the incidence among ethnic groups varies [1,2,4].

The problems arising from orofacial clefts are multifaceted, as the anatomical-morphological changes influence both functional criteria (breathing, speech, swallowing, hearing, craniofacial growth/development) and aesthetic and psychosocial aspects [2,3,5]. The rehabilitation protocol for these anomalies begins in the first few months of life [5] and involves a multidisciplinary team comprising professionals from various health specialties [4]. In all individuals, it is important to understand the possible etiological factors that caused the orofacial cleft [2], the behavioral aspects of the child and their family [6] and their social context [7], as well as determine a treatment plan that includes not only the aesthetic and functional rehabilitation of the child, but also good quality of life. Thus, the Special Issue “Current Research on Cleft Lip in Children” addresses the different themes of this subject, as well as presenting differentiated analyses on such a complex topic.

## 2. An Overview of Published Articles

Five manuscripts were approved for publication after a thorough editorial review process. The studies that comprise this issue are listed below:

The authors who contributed to this editorial come from different countries, such as Malaysia (1), Iraq (2), Poland (2), Italy (3), Switzerland (4 and 5) and Belgium (5), indicating a predominance of studies from European countries. This Special Issue includes interesting studies on the toxicity of a specific plant (1), mothers’ perceptions of treatments (2), quality-of-life analysis (3), the application of a three-dimensional (3D) digital flow (4) and the assessment of craniofacial growth and development (5). The contributions in this edition had the following aims and results:

Contribution 1 evaluated the toxicity of using a plant during the prenatal period in rats. The results suggested that certain doses of this plant could be potentially toxic to skeletal development during the fetal period.

Contribution 2 analyzed the perceptions of mothers in relation to the application of preoperative orthopedic appliances during the first years of life in child participants. The mothers had a positive perception because they recognized the clinical importance and were aware of the significant improvement in the children’s lives.

Contribution 3 assessed the quality of life of children/adolescents with and without orofacial clefts. Among the results found, it stands out that adolescents with cleft lip and palate report a negative impact on their self-image, highlighting the need for psychosocial monitoring.

Contribution 4 presented the application of a 3D digital flow to make orthopedic plates for newborns. This study shows that the 3D workflow has the potential to become standard in the rehabilitation protocol for individuals with orofacial clefts, as long as the costs of acquiring the necessary software, materials and equipment are overcome.

Contribution 5 analyzed whether bone grafting could restrict craniofacial growth and development. Among the data found, the omission of bone grafting did not improve growth in the 6–11-year-old age group.

The contributions in this Special Issue address essential issues that can influence etiological issues, the rehabilitation protocol and, consequently, the quality of life of children and adolescents. All of these topics are frequently studied due to the need to understand which etiological factors may be involved in the development of orofacial clefts, as this is a multifactorial issue [1,2]. Another issue is the rehabilitation protocol; it is known that there is currently no single rehabilitation protocol that motivates the professional studies of the rehabilitation team, because there are important criteria that must also be considered when analyzing the treatment protocol, such as the phenotype of the cleft lip and palate and its amplitude, the surgical technique applied, the age at which the individual underwent surgery, the skill of the plastic surgeon and also how the person themselves reacts to the treatment [8,9,10]. In a way, all of these approaches mentioned above influence the quality of life of both the children/adolescents and their parents/legal guardians; it is therefore essential to assess which points can influence well-being and how they can contribute to a significant improvement in this condition.

## 3. Final Considerations

Although cleft lip and palate continue to be subjects of ongoing research, there is a need for future research to be carried out in order to understand whether there are regional cultural or dietary habits that may contribute to the predisposition of congenital craniofacial anomalies. Additionally, addressing the knowledge gap related to the rehabilitation protocol is imperative to constantly seek updates that facilitate significant advancements in craniofacial development and growth. Finally, quality of life is another criterion that needs to be continually assessed, as cyberbullying is now a reality and its influence on the well-being of individuals must be taken into account. 

Thus, the studies presented in this Special Issue can be read not only by professionals directly involved in the treatment protocol for children with craniofacial anomalies, but also by people who can contribute in any capacity to the long and complex rehabilitation protocol. We hope and anticipate these articles will provide the motivation for future research into the various themes relating to cleft lip and palate.

## Data Availability

Not applicable.
